# Kinetic Modeling of *Saccharomyces cerevisiae* Central Carbon Metabolism: Achievements, Limitations, and Opportunities

**DOI:** 10.3390/metabo12010074

**Published:** 2022-01-13

**Authors:** David Lao-Martil, Koen J. A. Verhagen, Joep P. J. Schmitz, Bas Teusink, S. Aljoscha Wahl, Natal A. W. van Riel

**Affiliations:** 1 Department of Biomedical Engineering, Eindhoven University of Technology, Groene Loper 5, 5612 AE Eindhoven, The Netherlands; N.A.W.v.riel@tue.nl; 2 Lehrstuhl für Bioverfahrenstechnik, FAU Erlangen-Nürnberg, 91052 Erlangen, Germany; k.j.a.verhagen@tudelft.nl (K.J.A.V.); s.a.wahl@tudelft.nl (S.A.W.); 3 DSM Biotechnology Center, Alexander Fleminglaan 1, 2613 AX Delft, The Netherlands; joep.schmitz@dsm.com; 4 Systems Biology Lab, Amsterdam Institute of Molecular and Life Sciences, Vrije Universiteit Amsterdam, 1081 HZ Amsterdam, The Netherlands; b.teusink@vu.nl; 5 Amsterdam University Medical Center, University of Amsterdam, Meibergdreef 9, 1105 AZ Amsterdam, The Netherlands

**Keywords:** yeast, central metabolism, stress response, metabolic regulation, kinetic model, in vivo kinetics, parameter estimation, complexity, uncertainty, population heterogeneity

## Abstract

Central carbon metabolism comprises the metabolic pathways in the cell that process nutrients into energy, building blocks and byproducts. To unravel the regulation of this network upon glucose perturbation, several metabolic models have been developed for the microorganism *Saccharomyces cerevisiae*. These dynamic representations have focused on glycolysis and answered multiple research questions, but no commonly applicable model has been presented. This review systematically evaluates the literature to describe the current advances, limitations, and opportunities. Different kinetic models have unraveled key kinetic glycolytic mechanisms. Nevertheless, some uncertainties regarding model topology and parameter values still limit the application to specific cases. Progressive improvements in experimental measurement technologies as well as advances in computational tools create new opportunities to further extend the model scale. Notably, models need to be made more complex to consider the multiple layers of glycolytic regulation and external physiological variables regulating the bioprocess, opening new possibilities for extrapolation and validation. Finally, the onset of new data representative of individual cells will cause these models to evolve from depicting an average cell in an industrial fermenter, to characterizing the heterogeneity of the population, opening new and unseen possibilities for industrial fermentation improvement.

## 1. Introduction

*Saccharomyces cerevisiae* is a model organism in eukaryote cell research and the workhorse for the biotechnology industry [1]. In nature and the industrial setup, environmental perturbations act as stressing factors which challenge regulation of metabolic flux and can also lead to reduced performance in industrial applications [2]. For instance, perturbations in nutrient concentration often led to undesired outcomes such as lower process yields [3,4]. These perturbations alter intracellular fluxes in central carbon metabolism (CCM), the core pathways in the cell that process substrate into energy and building blocks [5], and to the products of biotechnology. To understand the functioning and dynamic response of CCM to glucose perturbations, multiple kinetic metabolic models have been developed.

Kinetic metabolic models are mathematical representations of a biological system that consider kinetic expressions such as rate constants. They describe the network structure, kinetic rate expressions and contain values for the parameters in these expressions [6]. Thus, these descriptions are well-suited to model time-dependent dynamics. A detailed explanation of the main components in a kinetic metabolic model can be seen in Box 1. Despite the progress attained with them, a consensus version with a full coverage of CCM has not yet been achieved.

This is explained by the fact that different works have approached this question with different data and tools. As a result, current models have a high degree of uncertainty, which represents an issue for the research community that strives to manage models and data following the FAIR principles [7,8,9]. Hence, a way to approach this problem is by reviewing the developed models in a systematic fashion, i.e., recall all the data and kinetic models that have studied it so far and to analyze their most relevant recent developments. Systematic reviews are well established in fields such as medicine, where they are aimed at critically and objectively synthesizing all available evidence regarding a specific topic, often accompanied by a meta-analysis leading to a consistent conclusion to a debated research question [10,11,12]. Therefore, in this work we aim to systematically survey the literature to determine which have been the advances, and which are the limitations, and opportunities in kinetic modeling of *S. cerevisiae* central carbon metabolism.

Box 1 **Kinetic metabolic models.**

Kinetic metabolic models are used to represent changing concentrations and reaction rates over time. For instance, this is useful to predict process yields or if a flux or intracellular concentration might reach dangerous levels. These models are described by a set of nonlinear ordinary differential equations (ODE), which assume ideally mixed compartments and neglect stochastic effects. The general form of these models is described by the following deterministic state space model (see [6,13,14] for more information):

(1) $$\frac{dx}{dt}\;=\;f\left(x\left(t,\theta \right),u\left(t\right),\theta \right)$$

(2) $$x\left(0\right)\;=\;x\left(\theta \right),\Delta t\left[t_{0},t_{f}\right]$$

where *x* represents a different state vector for every ODE, which usually consists of a metabolite concentration. *f* is a vector function where the change in state quantity is calculated at a given time point, using reaction rates which in turn depend on the states, the parameter vector θ and the system inputs *u*. The modeler must provide a simulation timespan Δt, initial states x(0), parameters θ and input *u*. The reactions composing these metabolic models are mostly catalyzed by enzymes. Therefore, intracellular reaction rates are often represented by Michaelis–Menten or Hill kinetics, and mass action when kinetic information is missing [15]. The parameter vector θ determining these reaction rates is composed by the following types of kinetic constants:

- Reaction rate constant, $V_{max}$: is determined by the catalytic constant and enzyme concentration ($V_{max}$ = $k_{cat}$·[E]). Thus, it varies as the cell changes its enzyme concentration in different environments.
- Catalytic constant, $k_{cat}$: indicative of how fast the reaction can go. Values in yeast models have been found as high as 5 · 10^2^ s^−1^ [16].
- Michaelis constant, $k_{m}$: indicative of the affinity of an enzyme for a metabolite. Values in yeast models are found in the range 10^−3^–10^1^ mM [16,17].
- Equilibrium constants, $k_{eq}$: Values are found in the range 10^−5^–10^3^ [17].
- Hill exponents, $n_{H}$: specific of reactions with hill kinetics.
- Additionally, enzymes can contain allosteric activation or inhibition

Models focusing on yeast glycolysis tend to contain around 25 species and 100 parameters [16,17,18], but the representations which have included other pathways in CCM [19,20] have increased as much as 42 states and 164 parameters.

## 2. The Literature Collected Point at an Increasing Complexity in Both Data and Models

**Figure 1:**
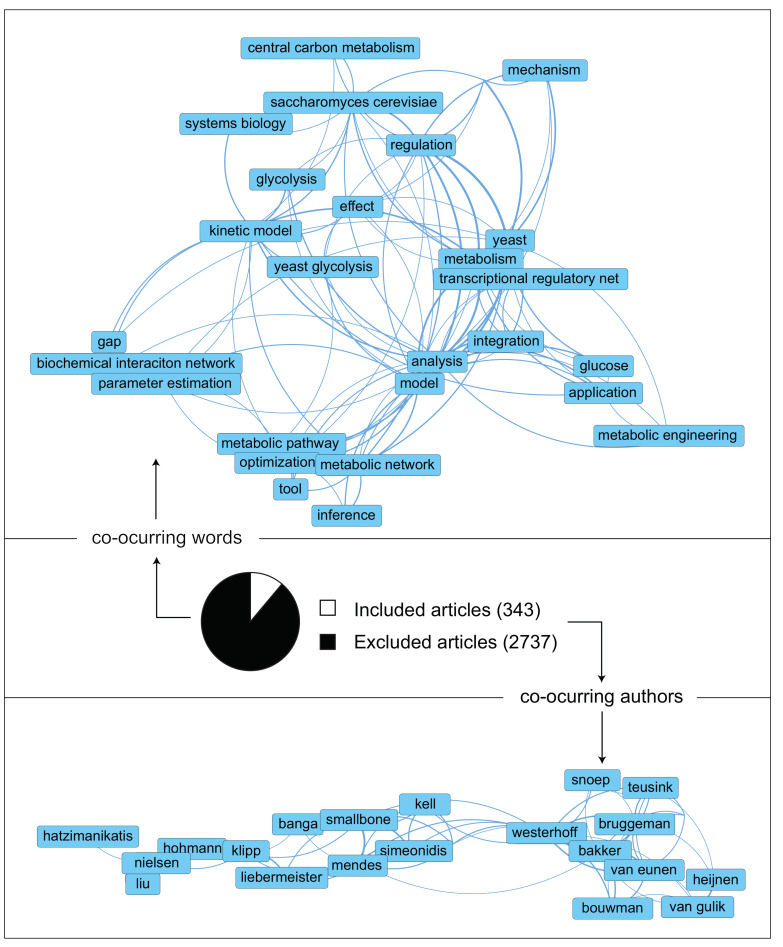
The literature collection presents the scientific landscape: (center) Articles examined in the reviewing process and fraction selected for this study. (top, bottom) Visualization of most co-occurring words and authors, respectively. in the titles of the selected articles. Obtained in VOSviewer. For the word map: counting method = binary, minimal occurrences = 5, terms selected = 100%. For the author map: counting method = full. The remaining setup was the default.

A pool of relevant literature articles could be obtained after the literature screening process described in Section 11. From the initial pool of 3080 articles, 2737 (close to 90% of them) could be discarded due to not meeting all the inclusion criteria or meeting any of the exclusion criteria (see Figure 1). A co-occurrence map of the most used words in the article titles showed that besides the topics specified in the search query, others that attracted attention in this field were pinpointed. These include topics such as glucose metabolism, parameter estimation, model optimization, inference, and integration. Then, a co-occurrence map of the authors highlighted authors with long experience in *S. cerevisiae* and/or development of kinetic models for systems biology.

**Figure 2:**
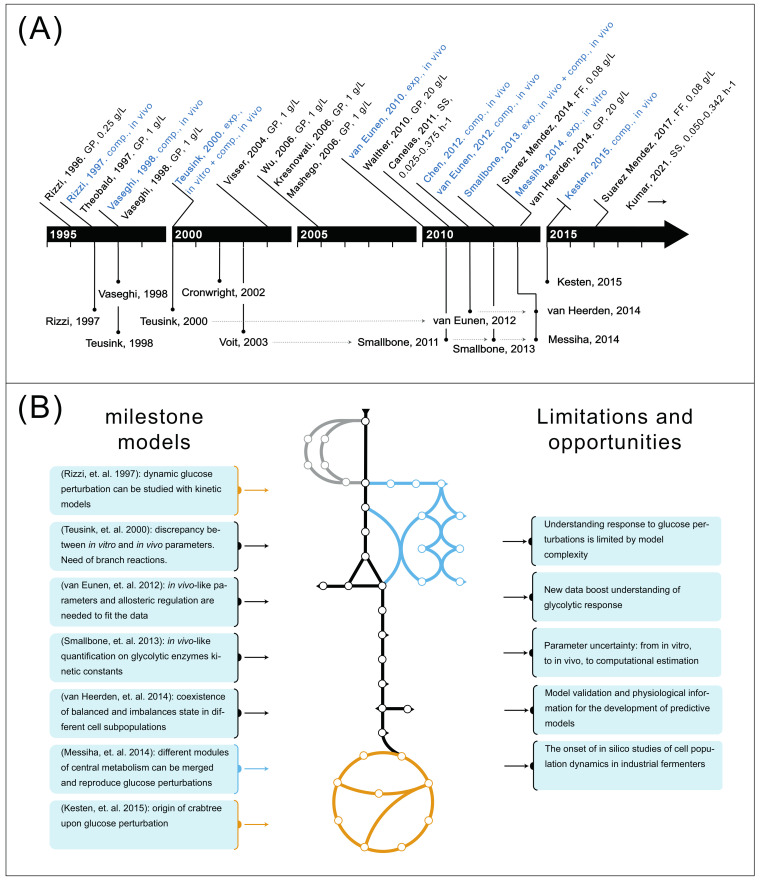
(**A**) A changing field presented by its literature: Above the timeline, from the literature pool of articles obtained in the systematic reviewing process, works which published new data sets are shown. These are displayed in black when the data consisted of intracellular metabolomics or fluxomics and in blue if it consisted of parameter values quantification. Below the timeline, newly developed metabolic models of pathways in central carbon metabolism are displayed. (**B**) (**left**) Contribution of the main models in the field, and (**right**) Limitations and opportunities for research. The simplified representation of CCM displayed in the middle is colored according to the how extensive is the coverage from the models in the left side. A complete trehalose cycle representation coupled to glycolysis (grey) does not exist yet.

Furthermore, the published models and datasets give an overview of how the field has recently developed (Figure 2). A first generation of models from 1997 to 2003 modeled different pathways contained in CCM but always in isolation [19,21,22,23,24] except for [19] which linked glycolysis and Tricarboxylic acid (TCA) cycle. These works made use of relatively small datasets developed for a single glucose perturbation (GP) experiment [21,25,26], and parameters were only measured experimentally in in vitro conditions [22].

From 2004 to 2014 the number of intracellular metabolites measured increased to almost a full glycolytic coverage [27,28,29,30], different intensity GP experiments were developed [18,31] and quantification of parameters was performed in in vivo conditions [16,32]. This allowed for the creation of a new generation and more predictive models which also linked glycolysis to trehalose cycle regulation [16,17,18], or the TCA cycle or pentose phosphate pathway (PPP) [20,33].

Finally, new experimental data have been generated since then. Although they remain unused for modeling development purposes, these data consist of metabolic concentrations but also biochemical flux measurements data (often regarded as metabolomics and fluxomics, respectively) at different growth rates (labeled here as steady states, or SS) [34,35], and repetitive cycles with moderate changes in substrate availability (also known as feast–famine (FF) experimental setup) [36,37]. Detailed overviews of the models developed, experimental metabolomic/fluxomic data sets and parameter quantification assays can be seen in Table 1, Table 2 and Table 3, respectively, and are discussed in the coming sections.

## 3. Glycolytic Response to Glucose Perturbations in Yeast Fermentations

*Saccharomyces cerevisiae* is one of the most used microorganisms in biotechnology. *S. cerevisiae* is a prominent cell factory involved in food, beverages, and biofuels industries [38,39]. On top of its favorable physiology and robustness, genetic engineering has allowed to introduce new pathways and improve existing ones, generating new strains that have widened its range of applications [1,40]. Nonetheless, scaling up to commercial production is a challenging stage in which developed strains may emerge as inefficient [41]. Long circulation times and nonideal mixing result in substrate gradients in the industrial fermenter, affecting most cell factories, including *S. cerevisiae* [3,4,42,43]. The yeast cell sees these gradients as stressing factors to which it continuously adapts, often deteriorating process yields and giving relevance to the development of stress tolerant strains [2].

Extracellular substrate gradients alter intracellular fluxes in CCM. Carbon flux shifts between the different pathways composing CCM during these temporal transitions [5]. This can become a challenge for the cell, which struggles to keep the different pathways composing CCM balanced [44], as was shown in [18] for a yeast strain with a defective trehalose cycle, where sudden exposure to a high glucose concentration resulted in growth arrest. Glycolysis is found at the core of this network. This pathway digests intracellular glucose into pyruvate and produces energy in the form of ATP and glycolytic intermediates that support anabolic reactions [45].

How glycolysis contributes to the metabolic processes inside the cell depends on multiple factors. The presence or absence of oxygen determines if pyruvate is used for respiration or fermentation [46,47]. Still, this conspicuously simple explanation is challenged at high-substrate concentrations, where the maximum respiratory capacity is reached and fermentation takes place even if oxygen is present [48,49], in what is known as ‘overflow metabolism’ or Crabtree effect [50]. In addition, the substrate that is used as carbon source (such as glucose or fructose) and the ability of a strain to metabolize it also affects glycolytic kinetics and process yields [51,52,53,54,55,56,57]. Furthermore, the cellular state determines how glycolytic intermediates are used as biomass precursors [45,58]. For instance, at changing growth rates, different usage of these precursors can be observed [34,59]. Finally, availability of cofactors cannot always be taken for granted. A higher substrate uptake rate might be an evolutionary advantage, but it results in a demand for NADH recycling that respiration cannot achieve and thus fermentation becomes active [60,61].

The response of glycolysis to dynamic glucose perturbations is controlled by different regulatory layers. The first mechanism is the storage of glycogen and trehalose when glucose uptake exceeds the glycolytic processing capacity [62]. On top of this, allosteric and post-translation regulation take place [63]. Hexokinase (HXK) is allosterically inhibited by trehalose-6-phosphate (T6P), pyruvate kinase (PYK) is activated by fructose-1,6-bis-phosphate (FBP) and multiple metabolites act on phosphofructokinase (PFK) [27,64,65]. Simultaneously, the cAMP-protein kinase A (PKA) pathway is activated upon glucose perturbation and starts a regulation cascade in CCM [66] and possible targets for Post-Translational Modifications (PTMs) have been found in multiple enzymes along the CCM [67]. Finally, to adapt to different growth conditions, yeast cells use different enzyme isoforms. For instance, hexokinases and glucokinases are balanced to adapt to different glucose concentrations [68] and the regulation of intracellular pH is compartment-specific, carried out by different ATPases [69].

## 4. The Development of Metabolic Models Has Resulted in Understanding of Key Glycolytic Properties

Many breakthroughs in metabolic modeling used genome scale models. Nonetheless, stoichiometry alone does not define function and the response to glucose perturbations is a dynamic process where stoichiometry cannot explain mechanisms that act at different time scales or the appearance of bistability, among others [6,70]. As a result, kinetic models enable a deeper understanding of glycolytic properties. Due to the abundant data available for *S. cerevisiae* fermentations, models of the glycolytic networks have reached a high level of maturity for this organism.

The first kinetic models developed focused on understanding glycolytic oscillations in nongrowing yeast cells [23,71,72,73,74,75,76,77,78]. Most enzymatic reactions were lumped into a few (except [23,77]) but they acknowledged the important role of enzyme PFK and showed sensitivity to different glucose, oxygen, and acetaldehyde concentrations. Later works focused on understanding control properties and glycolytic response upon a single glucose perturbation experiment [19,79,80,81,82] and thanks to a progressive increase in experimental data available, more detailed models were developed [16,17,18,22]. Much of the focus was on understanding how mutant strains lacking a functional trehalose cycle would undergo growth arrest upon the glucose perturbation [62,64]. This was found to be due to a glycolytic imbalance between upper and lower glycolysis and attributed first to an ATP turbo metabolism [82]. Later, ref. [18] explained the role that the trehalose cycle plays in the glycolytic response and highlighted how the intracellular concentrations of metabolites at a given time point modulate the outcome.

In this process, models have become more interconnected with other pathways, allowing for a more complete understanding of the glycolytic response. Ref. [22] introduced glycolytic byproduct branch reactions that were necessary to reproduce the steady state. Other works modeled pathways that are directly linked to yeast glycolysis. For instance, detailed descriptions of the glycerol synthesis, trehalose cycle and PPP were developed in [21,24,83], respectively. Later, a PPP model was connected to glycolysis in [33], and another model of glycolysis together with TCA was developed in [20]. These networks were used to understand the control properties of glycolysis, pointing to glucose transporter (GLT) and PFK for being the enzymes with the highest controlling coefficients [79,80,84,85,86] and to study the effect of genome duplications [87]. For a complete overview of metabolic models developed to understand dynamic perturbations, see Table 1.

Furthermore, the regulation exerted by cofactors has gradually become more evident, resulting in a more complex understanding of glycolysis. The depletion of inorganic phosphate concentration that was shown to be crucial in [18] had been overlooked in previous works where it was assumed to be constant over time. Simultaneously, the sum of adenosine nucleotides has been assumed to be a conserved moiety [16] but under some experimental conditions this is not the case [31,88], which can be relevant considering that controlling enzyme PFK is allosterically regulated by ATP and AMP.

**Table 1:** Properties of *S. cerevisiae* models developed to understand dynamic glucose perturbation response: glycolysis (GLYCO), tricarboxylic acid cycle (TCA), pentose phosphate pathway (PPP), trehalose cycle (TRE). Number of ‘+’ sign according to how advantageous the property is. Cofactor conservation moieties are sumAXP and sumNADX. N/A when reactions were not modeled, or data were not shown in article. Refs. [17,20] fitted different parameter sets to multiple data sets. Other models used a unique parameter set. From the literature pool of articles obtained in the systematic reviewing process, only the works which include glycolysis are displayed.

	Rizzi et al. [19]	Teusink et al. [82]	Teusink et al. [22]	van Eunen et al. [17]
Contribution to glycolytic understanding	Dynamic models can accurately describe glucose perturbation.	ATP surplus can cause the observed overactivation of initial glycolytic steps in DTps1 mutant strains.	In vivo behavior cannot be predicted with in vitro kinetics.	Implementation of allosteric regulation and in vivo measured parameter values is necessary to reproduce GP data.
GLYCO	Individual + branch reactions (++)	Lumped reactions (+)	Individual + branch reactions (++)	Individual + branch reactions (++)
TRE	N/A	N/A	N/A	T6P regulation (+)
TCA	Individual reactions (++)	N/A	N/A	N/A
PPP	N/A	N/A	N/A	N/A
Cofactors	Conservation moiety (+)	Conservation moiety (+)	Conservation moiety (+)	Conservation moiety (+)
Parameters	Computational, in vivo (++)	Computational, toy model (+)	Computational, in vivo (++)	Experimental and computational, in vivo (++)
Data	Single GP experiment (++)	Single GP, toy data (+)	SS data point (+)	Single GP experiment and multiple SS (+++)
	** Smallbone et al. [16]**	** Van Heerden et al. [18]**	** Messiha et al. [33]**	** Kesten et al. [20]**
Contribution to glycolytic understanding	Broad quantification of enzymatic kinetic constants in in vivo-like conditions.	Glycolytic dynamics combined with cell heterogeneity determine cell fate.	Feasibility of constructing larges network models by merging smaller pathway models.	Cooperativity PYK-PYR and ADH-PDH bypass play a major role in the onset of the Crabtree effect.
GLYCO	Individual + branch reactions + isozymes (+++)	Individual + branch reactions (++)	Individual + branch reactions (++)	Individual + branch reactions (++)
TRE	N/A	T6P regulation (+)	N/A	N/A
TCA	N/A	N/A	N/A	Individual reactions (++)
PPP	N/A	N/A	Individual reactions (++)	N/A
Cofactors	Conservation moiety (+)	Conservation moiety + dynamic Pi (++)	Conservation moiety (+)	Conservation moiety (+)
Parameters	Experimental, in vivo (++)	Experimental, in vivo (++)	Experimental, in vivo (++)	Computational, in vivo (++)
Data	N/A	Single GP experiment (++)	Single GP experiment (++)	Single GP experiment (++)

## 5. From Glycolysis to Central Carbon Metabolism: Understanding Response to Glucose Perturbations Is Limited by Model Complexity

Development of kinetic models of metabolism has often been constrained to small systems. In *S. cerevisiae* models, each next step forward in the understanding of glycolysis encountered a new limitation due to the inherent complexity of the pathway.

Models studying glycolytic oscillations or single GP experiments led to an in-depth analysis of glycolytic dynamics, but to understand central carbon metabolism performance, more pathways than only glycolysis must be considered. For instance, a significant fraction of glucose-derived carbon is taken up at different points in glycolysis [34]. To account for this, a relatively simple option is to implement branches or sink reactions (developed for *Escherichia coli* in [59]). This led *S. cerevisiae* models to reproduce steady state where imbalance had been mistakenly predicted [22]. Still, dynamic regulation of storage metabolism is more complex than a sink reaction [18,37] and later models gradually added complexity to the trehalose cycle kinetics to avoid the imbalance from happening upon dynamic perturbation [17,18]. A similar situation could happen for other closely linked pathways such as the TCA or PPP, which have mostly been lumped into a single reaction, even though a few exceptions exist [19,20,21,33]. Simultaneously, other approaches such as linlog kinetics have aimed at attaining high model complexity but with simplified expressions using less parameters [89,90,91].

Furthermore, factors such as growth rate, compartmentation, or transport of metabolites other than glucose, regulate glycolytic response but have barely been considered. First, the growth rate determines how sink reactions behave [34], but most models focus only on a unique growth rate of 0.1 h^−1^. Since the effect of this variable has not been explicitly considered, models simulating different growth rates had no other alternative than to fit a different parameter set each time [17]. Second, compartmentation and transport reactions have barely been considered and, for instance, this is relevant in trehalose regulation since it is known to accumulate in compartments other than the cytosol [92,93]. Third, transport of metabolites such as gases oxygen (O_2_) and carbon dioxide (CO_2_) could allow models to explain differences between respiratory and fermentative behavior [27,30,50] but neither has been implemented.

On top of this, other variables affect individual enzyme kinetics, and have neither been considered. First, cytosolic pH decays upon extracellular glucose perturbation, affecting multiple intracellular processes, including enzyme kinetics [69,94]. Second, PTMs are a fast response mechanism and multiple target sites have been found throughout CCM [95]. Third, different enzyme isoforms are expressed under different growth regimes. Examples of this are the differential expression of GLK/HXK and Glyceraldehyde 3-phosphate dehydrogenase (GAPDH) genes ([96] and [97], respectively).

Finally, a key challenge is the representation of variables that are not part of the carbon flux, such as cofactors. Most models have kept them constant or adopted moiety conservation cycles [98], such as the sum of intracellular adenine nucleotides ([ATP] + [ADP] + [AMP] = [AXP]) or inorganic phosphate [17]. Nonetheless, under intense glucose perturbations, both variables behave in a dynamic manner [19,26,31,99] and alter glycolytic response. An example of this is the ATP paradox, which occurs when ATP and the sum of adenine nucleotides transiently decay [100]. Understanding cytosolic Pi as a dynamic variable and implementation of import from the vacuole turned out to be central in understanding the glycolytic imbalance [18]. Although the availability of Pi was essential for lower glycolysis progression via GAPDH [18], adenine nucleotides exert allosteric regulation on the important controlling enzyme PFK [101].

## 6. New Intracellular Metabolomic and Fluxomic Data Boost Understanding of Glycolytic Response

Scale-down approaches have been developed to understand long-standing problems in industrial bioreactors. Although this has granted valuable knowledge, essential intracellular properties such as in vivo fluxes and kinetics have been captured with only limited resolution, constraining model development. In fact, this has become one of the main challenges in the development of high quality predictive kinetic models, since often multiple variables, such as transcriptomics, metabolomics and fluxomic data, interact to result in the final response [6].

Early works aimed to understand glycolytic oscillations did so with small datasets, reducing their range of implementation. On most occasions only extracellular data such as growth and nutrient exchange rates was available [76] or a few metabolites at most [75], until in vivo quantification of metabolite concentrations and fluxes became a common practice, where most cofactors, glycolytic intermediates and rates were simultaneously observable [22]. Later, a standardized dynamic glucose perturbation experimental setup with CEN-PK yeast strains was adopted (see Table 2). This consisted of chemostat growth at dilution rate of 0.1 h^−1^, followed by an external glucose perturbation, where extracellular concentration increased to 1 g L^−1^. These stimulus response experiments were used to infer more physiological patterns [26] and the use of Nuclear Magnetic Resonance (NMR) and Mass Spectroscopy (MS) techniques made a wide range of intracellular metabolites measurable. From only a few glycolytic concentrations, datasets gradually grew to include most metabolites in glycolysis, the trehalose cycle, the TCA cycle, and the PPP. Adenine nucleotides and NAD:NADH ratio have also been made a standard and other nucleotides and amino acids which are affected by carbon uptake dynamics are quantified in the most recent publications.

**Table 2:** Glucose perturbation experiments in *S. cerevisiae* with intracellular metabolome quantification: Stirred tank reactors (STR) operated in chemostat. Shake flasks (SF) in batch conformation. Metabolite pools: glycolysis (GLYCO), tricarboxylic acid cycle (TCA), pentose phosphate pathway (PPP), trehalose cycle (TRE), nucleotides (NUC), Amino acids (AAs). Even though intracellularly localized, variables measured were whole cell, and exceptions are pointed. From the literature pool of articles obtained in the systematic reviewing process, the works displayed measured experimentally intracellular variables such as metabolite concentrations or fluxes. Literature is ordered by glucose input regime.

	Rizzi et al. [25]	Theobald et al. [26]	Vaseghi et al. [21]	Visser et al. [27]
Glucose input regime	Glucose-limited to glucose pulse (0.25 g L^−1^)	Glucose-limited to glucose pulse (1 g L^−1^)	Glucose-limited to glucose pulse (1 g L^−1^)	Glucose-limited to glucose pulse (1 g L^−1^)
Experimental setup	30 °C, pH5, aerobic, D = 0.1 h^−1^, STR, direct sampling	30 °C, pH5, aerobic, D = 0.1 h^−1^, STR, direct sampling	30 °C, pH5, aerobic, D = 0.1 h^−1^, STR, direct sampling	30 °C, pH5, aerobic, D = 0.05 h^−1^, STR, BioScope sampling
Duration	500 s	180 s	180 s	80 s
Strain	CBS 7336 (ATCC 32167)	CBS 7336 (ATCC 32167)	CBS 7336 (ATCC 32167)	CEN.PK113-7D
Measurement technique	Enzymatic assay	Enzymatic assay: metabolites, NAD(H) HPLC: adenine nucleotides	Enzymatic assay: metabolites, NAD(H)	Enzymatic assay: ATP, NADX and G6P MS: glycolytic intermediates
Intracellular variables measured	**GLYCO**: G6P.	**GLYCO**: G6P, F6P, FBP, GAP, 3PG, PEP, PYR. **NUC**: NAD(H), AXP (whole cell and cytoplasmic). Pi.	**GLYCO**: G6P, F6P. PPP: 6PG. **NUC**: NADP(H).	**GLYCO**: G6P, F6P, G1P, FBP, 2GP+3PG, PEP, PYR. **NUC**: ATP, NADX.
Glucose input regime	Glucose-limited to glucose pulse (1 g L^−1^)	Glucose-limited to glucose pulse (1 g L^−1^)	Glucose-limited to glucose pulse (1 g L^−1^)	Trehalose-limited to glucose pulse (20 g L^−1^)
Experimental setup	30 °C, pH5, aerobic, D = 0.05 h^−1^, STR, BioScope sampling	30 °C, pH5, aerobic, D = 0.05 h^−1^, STR, BioScope sampling	30 °C, pH5, aerobic, D = 0.05 h^−1^, STR, direct sampling	30 °C, pH4.8, aerobic, SF, direct sampling.
Duration	180 s	180 s	300 s	30 min
Strain	CEN.PK113-7D	CEN.PK113-7D	CEN.PK113-7D	BY4741
Measurement technique	MS	Enzymatic analysis: NAD(H) MS	MS	MS
Intracellular variables measured	**GLYCO**: G6P, F6P, FBP, 2/3PG, PEP, PYR. **TCA**: ISOCIT, FUM, MAL, AKG, SUC. **PPP**: 6PG. **TRE**: G1P, T6P, TRE. **NUC**: AXP, NADH:NAD ratio.	**GLYCO**: G6P, F6P, F1,6P2, F2,6P2, 2/3PG, PEP. **TCA**: ISOCIT, AKG, SUC, FUM, MAL. **PPP**: 6PG. **TRE**: G1P, T6P. **NUC**: AXP, NADH:NAD ratio.	**GLYCO**: G6P, F6P, F1,6P2, F2,6P2, 2/3PG, PEP. **TCA**: ISOCIT, AKG, SUC, FUM, MAL. **PPP**: 6PG. **TRE**: G1P, T6P. **NUC**: AXP, NADH:NAD ratio. AAs.	**GLYCO**: G6P, F6P, FBP, G3P, 2/3PG, PEP. **TCA**: AKG, MAL. **PPP**: 6PG, R5P, R1P. **TRE**: T6P, G1P. **NUC**: ATP, ADP, AMP, IMP, INO, HYP, GTP, GDP, GMP.
	**Van Heerden et al. [18]**	**Suarez-Mendez et al. [36], Suarez-Mendez et al. [37]**	**Canelas et al. [34]**	**Kumar et al. [35]**
Glucose input regime	Glucose-limited to glucose pulse (20 g L^−1^)	Glucose-limited to feast–famine cycles (0.08 g L^−1^ max.)	Glucose-limited. Dilution rates from 0.025 to 0.375 h^−1^	Glucose-limited. Dilution rates from 0.050 to 0.342 h^−1^
Experimental setup	30 °C, pH5, aerobic, D = 0.1 h^−1^, STR, BioScope sampling	30 °C, pH5, aerobic, D = 0.1 h^−1^, STR, direct sampling	30 °C, pH5, aerobic, STR, direct sampling	30 °C, pH5, aerobic, STR, direct sampling
Duration	340 s	400 s	N/A (ss)	N/A (ss)
Strain	CEN.PK113-7D	CEN.PK113-7D	CEN.PK113-7D,mtlD1	CEN.PK113-7D
Measurement technique	MS Reaction rates calculated by piecewise affine approximation (13C data)	MS Reaction rates calculated by piecewise affine approximation (13C data)	MS Reaction rates calculated with a stoichiometric model	MS
Intracellular variables measured	**GLYCO**: G6P, F6P, FBP. **TRE**: G1P, UDPG, T6P, TRE. **PPP**: 6PG. **NUC**: AXP, cAMP, UXP, GXP. **Fluxes** within glycolysis and trehalose cycle.	**GLYCO**: G6P, F6P, FBP, G3P, GLYC, DHAP, GAP, 2PG, 3PG, PEP, PYR. **TCA**: CIT, FUM, ISOCIT, MAL, AKG, SUC. **PPP**: 6PG, E4P, R5P, RBUP5, S7P, X5P. **TRE**: G1P, UDPG, T6P, TRE. **NUC**: AXP. **Fluxes** within glycolysis and trehalose cycle.	**GLYCO**: G6P, F6P, FBP, F26BP, G3P, DHAP, GAP, 2PG, 3PG, PEP, PYR. **TCA**: CIT, FUM, ISOCIT, MAL, OAA, SUC. **PPP**: 6PG, E4P, R5P, RBUP5, S7P, X5P. **TRE**: G1P, T6P, TRE. **NUC**: AXP, UXP, cAMP, NAD:NADH ratio. AAs. **Fluxes** within glycolysis.	**GLYCO**: G6P, F6P, FBP, G3P, DHAP, 2/3PG, PEP, PYR. **TCA**: CIT, FUM, OAA, ISOCIT, MAL, AKG, SUC. **PPP**: 6PG, R5P, RBUP5, S7P. **TRE**: G1P, UDPG. **NUC**: AXP, GXP, IXP, TXP, UXP, dAXP, dGXP, dUXP. AAs.

Nevertheless, several issues limit quantification of intracellular variables. First, accurate quantification becomes challenging due to the need for quenching [103], intracellular/extracellular separation [104] or rapid sampling [102,105] which is especially relevant when variables have low concentrations and high turnover rates such as the ratio NAD:NADH [106]. Second, some variables are not always measured, hindering comparison between experiments. One example is the feat of nucleotides when the ATP paradox takes place. Although in [31] it was observed that the missing metabolites were being stored in the inosine salvage pathway, their measurement is still not a standard practice. Third, the lack of tools to measure compartment-specific concentrations limits our understanding of the interplay between cytosol, mitochondria, and vacuole. This is a relevant matter since the thermodynamic environment encountered in a specific compartment can alter reaction kinetics, to what cofactors can be notably sensitive [107].

Conversely, recent years have witnessed key technological advances that extend and improve our ability to quantitatively monitor relevant variables. The use of internal ^13^C standards enabled more accurate quantification of intracellular metabolite concentration [108]. ^13^C substrate tracing enabled the determination of steady-state fluxes [109] and it has been extended to monitor glucose perturbation [18] and several steady states at different growth rates [35]. Simultaneously, a new experimental approach has been developed: dynamic feast–famine cycles [92]. These repetitive cycles resemble more closely the environment that yeast cells experience in the industrial fermenter and extensive datasets are now available, even though not yet used for the purpose of model development [36,37]. Furthermore, proteome quantification can help understand how fermentative and respiratory capacities evolve with growth rate [60,110]. At changing growth rates, the relative protein expression is different for each glycolytic enzyme and this dependency can be used to constraint the models by adjusting kinetic constants accordingly. This type of approach can be extended to also quantify PTMs which modulate enzyme activity of central metabolism [95]. Finally, some tools have started to shed light on developments which take place inside compartments. Promising technologies such as equilibrium-based reactions, FRET sensors [107,111,112,113], microfluidics and other single-cell technologies could potentially be used to measure variables inside the mitochondrion, for instance.

## 7. Parameter Uncertainty: From In Vitro, to In Vivo, to Computational Estimation

Uncertainty is a recurring obstacle in the development of kinetic metabolic models [114]. It can be categorized in two types: epistemic, when it can be reduced by gathering more data or refining the model, or aleatoric, when the uncertainty is an inherent feature of the system [115], such as the case of stochasticity associated with biochemical systems containing low concentrations of many species [116]. Although the network stoichiometry of CCM is well known and its allosteric or post-translational regulation can be experimentally measured, parameters are hard to quantify. Therefore, parametric uncertainty is a major challenge when dealing with large-scale kinetic networks [117]. For *S. cerevisiae* CCM models, parametric uncertainty was initially aleatoric, as in vivo values were not quantifiable, but the recent decades have seen important progress in this area.

In *S. cerevisiae* models, parameter values have been often quantified in vitro and in conditions that maximize the activity of each individual enzyme but do not resemble the cellular environment [32]. When embedding these parameters in a model, simulations often led to unrealistic behaviors [22]. Furthermore, only a subset of parameters can be directly measured [15]. Consequently, a great effort was directed to developing a standardized assay media that resembles the yeast cytosol and many glycolytic parameters have been redetermined in these in vivo-like conditions [32] and implemented in kinetic models [16,17]. An overview of publications where kinetic parameters were estimated can be seen in Table 3.

**Table 3:** Overview of studies that quantify central metabolism kinetic constants in *S. cerevisiae*: Only works that aimed to study glycolysis as a system are shown. Prior works that studied glycolytic oscillations or individual enzymes are not displayed. Experimental data that did or did not resemble the yeast cell cytosol are referred to as in vivo or in vitro, respectively. Pathways parameterized are glycolysis (GLYCO), tricarboxylic acid cycle (TCA), pentose phosphate pathway (PPP), trehalose cycle (TRE). From the literature pool of articles obtained in the systematic reviewing process, the works displayed estimated parameter values. Publications [16,22] appear in two columns because they simultaneously used two different parameter estimation methods to quantify the same and different kinetic constants type, respectively. Literature is ordered by parameter estimation method.

	Teusink et al. [22]	Messiha et al. [33]	van Eunen et al. [32]	Smallbone et al. [16]
Parameter estimation	Experimental, in vitro	Experimental, in vitro	Experimental, in vivo	Experimental, in vivo
Type of constant	$V_{max}$	$K_{m}$, $K_{cat}$	$V_{max}$	$K_{m}$, $K_{cat}$
Pathway	GLYCO	PPP	GLYCO	GLYCO
Experimental condition	Enzymatic assay. Enzyme-specific	Enzymatic assay. Enzyme-specific	Enzymatic assay. Cytosol-like	Enzymatic assay. Cytosol-like
	** Rizzi et al. [19]**	** Vaseghi et al. [21]**	** Teusink et al. [22]**	** van Eunen et al. [17]**
Parameter estimation	Computational, in vivo	Computational, in vivo	Computational, in vivo	Computational, in vivo
Type of constant	$V_{max}$	$V_{max}$	$V_{max}$	$V_{max}$, $K_{m}$
Pathway	GLYCO, TCA	PPP	GLYCO	GLYCO (GAPDH)
Experimental condition	GP (1 g L^−1^)	GP (1 g L^−1^)	SS (0.1 h^−1^)	GP (1 g L^−1^)
	** Chen et al. [118]**	** Smallbone et al. [16]**	** Kesten et al. [20]**	
Parameter estimation	Computational, in vivo	Computational, in vivo	Computational, in vivo	
Type of constant	$V_{max}$	$V_{max}$	$V_{max}$, $K_{m}$	
Pathway	GLYCO	TRE	GLYCO, PPP, TCA	
Experimental condition	SS (0.1 h^−1^)	SS (0.1 h^−1^)	Either SS (0.1 h^−1^) or GP (1 g L^−1^)	

Despite this improvement, the accuracy of the parameter values was rarely estimated and enzymes were studied in isolation, rather than from a systems perspective. A common practice to deal with this problem is to re-estimate a subset of the parameters from a complete glycolysis model to fit in vivo data, namely metabolomics, frequently in a Maximum Likelihood Estimation (MLE) problem [17,19,20,23,77,79,86]. Still, only a few works quantified the differences between in vitro and in vivo parameters [22,119].

Nonetheless, there are opportunities to reduce parametric uncertainty in the near future. Kinetic constants quantified in cytosol-like conditions have only been used in a few works [16,17]. In addition, a considerable part of the data generated in recent years has not yet been used for validation, nor quantification of kinetic constants. In the last decade, extensive metabolomics and fluxomics datasets have been generated [18,34,36] and proteomics data are growingly available [60,120,121]. These data can now be used to extend our knowledge in central metabolism modeling, for instance by re-fitting parameter values or validating model simulations, even though it is rarely available in public repositories.

Moreover, computational quantification of kinetic constants allows the performance of a feasibility check by comparing experimental and estimated parameters [6,122], even though the scale of the network can become a burden since the estimation problem can be underdetermined. To deal with this issue, the so-called divide-and-conquer approach could be beneficial since it exploits a decomposition of the global estimation problem into independent subproblems, which are easier to deal with as the problem scale and variables involved are less [122,123]. If the subproblems are still ill-conditioned, regularization can be implemented to supplement the MLE problem with additional biological information [13]. A commonly used approach with dynamic models is L1 or Tikhonov regularization, which adds a penalty on parameters that deviate from a specific value, favoring the estimates that resemble experimental measurements [124,125,126,127]. Furthermore, to deal with big scale kinetics models, multiple toolboxes have been developed that assist in the development and analysis of this large-scale models [128,129,130,131], and benchmarking studies have evaluated their performance in different setups [14,132], which will help the modeler select the tool that is best suited for a particular problem.

## 8. Model Validation and Inclusion of Physiological Variables Regulating Glycolysis Are Needed for the Development of Predictive Models

To establish the credibility of a computational model, a pipeline of verification, validation, and uncertainty quantification (VVUQ) is followed [133]. Guidelines are also available to make research findable and reproducible [134,135]. Model verification mostly concerns with proper modeling and technical practices while validation is performed by means of reproducing physiological properties and new experimental data and robustness studies [6,136,137].

A common validation practice in *S. cerevisiae* models has been to construct models with parameter values measured in experimental assays and then simulate metabolomics in vivo data. If predictions did not match, either a subset of parameters was re-estimated, or this was used to generate new hypotheses or as an indication of uncertain areas in the model that needed improvement [17,19,20,22,79,81]. Proper model physiology has been often evaluated by simulating gene duplication or mutant strains [18,82,87] or studying its metabolic control properties [79,80,85,86].

Nevertheless, the experimental data used to validate models has been restricted to a single experimental setup: A glucose perturbation from 0.01 to 1 g L^−1^ of glucose concentration in a steady-state culture at dilution rate of 0.1 h^−1^ (Table 1). Despite this, it has been achieved for other pathways external to CCM where different nutrient pulses could be reproduced with the same model [119,123] and the above-mentioned feast–famine experiments and steady states at different dilution rates could provide new insights if modeled. Still, to achieve this, current models need to be expanded to represent necessary physiological variables.

For instance, to simulate steady states at different dilution rates [34], the effect of growth rate must be considered. Current models simulate nongrowing yeast cells, but in the industrial setup this is more often the exception than the rule. When growth rate changes, so do the amounts of glycolytic intermediates that are taken for biomass synthesis, the use of cofactors, and the predominance of respiration or fermentation, what could explain the condition-specific parameters in [17]. To account for this, one option is to implement a sink reaction term in the mass balance of each glycolytic intermediate that is taken up for biomass synthesis, as has been implemented in *E. coli* models [59]. Moreover, changing growth rates also meant that the sum of adenosine nucleotides was not kept constant [34], a phenomenon that can become impactful for glycolytic kinetics. Even though this could be partially explained by growth associated maintenance and non-growth associated maintenance, these physiological variables have not been considered yet.

Furthermore, implementing transport rates for substrates other than glucose and (by)products would not only validate the model, but would also allow the use of new data and simulate conditions that are important in the bioreactor production setup [6]. Process yield is altered depending on the carbohydrate substrate (fructose, galactose, maltose or sucrose, among others) [51,52,53,54,56]. Even though its implementation should not present a computational burden since only transport or isomerization reactions must be added in the model, only sugar uptake kinetics were implemented in black-box models [51,52,53,55,56,57] (but see exception for galactose in [54]). Moreover, byproduct exchange of gases, routinely measured [27,34,36], could also be accounted for as in [119]. For instance, exchange rates qO_2_, qCO_2_ and RQ ratio could help explain how availability of oxygen limits respiratory flux in both industrial [43] and lab-scale yeast fermentations [47]. To account for CO_2_, its production by pyruvate decarboxylase and the TCA cycle has been implemented but the only model constraint has been glucose concentration [18,19,20,22]. Another relevant example are fermentation (by)products such as ethanol. Even though it is known to be inhibitory above a concentration threshold [27,138], it has rarely been considered.

Finally, the yeast cell experiences gradients of pH and temperature in the industrial bioreactor that can have a severe impact on the fermentation. Since the effect of these environmental variables in the cytosol can be widespread to virtually all enzymes, modeling has been less detailed and has used cellular black-box models [139,140,141] rather than specific enzymatic kinetics. Nonetheless, for other organisms the effect of pH in glycolytic enzymes has been implemented [142,143] and this could also become the case for yeast models considering the increase in experimental data [144,145].

## 9. The Onset of In Silico Studies of Cell Population Dynamics in Industrial Fermenters

The ideal mixing assumption rarely holds in large-scale bioreactors, resulting in substrate gradients and lower process yield [3,4,42]. Therefore, understanding the interplay between the yeast cell and its surrounding environment becomes paramount to optimize the bioprocess performance. Obtaining experimental data and process optimization at the industrial fermentation scale is challenging and costs can become prohibitive. As a result, downscaling experiments have been developed to aid in strain selection [26,36]. In this process, the conditions in the industrial fermenter must be properly understood to develop appropriate downscaling setups.

One approach has been to study the heterogeneity within cell subpopulations. Single-cell analysis devices have been developed and the changes in glucose concentration have been shown to be comparable to the perturbations experienced by the cell in the industrial fermenter [146]. Furthermore, these studies have shown how cell-to-cell heterogeneity can be prevalent for many physiological variables such as growth rate, morphology, gene expression and cell viability, even if the extracellular environment is the same [147,148,149,150]. This is even more relevant considering that research in [18] showed how intracellular metabolic concentration heterogeneity could result in growth cell arrest for some cells in a population.

Despite the valuable insights that these downscaling experiments have provided, they are limited to study only one experimental design at a time, while in the industrial bioreactor multiple metabolic regimes are present and simultaneously contribute to the process yield. To understand the full picture, in silico modeling studies are essential. For instance, computational fluid dynamics (CFD) simulations have been developed to simulate the different gradients that each cell in the population experiences. Each of these simulations is referred to as a *lifeline* [4] and has been implemented to study oxygen and glucose gradients in *S. cerevisiae* fermentations [43,151]. From the different lifelines, substrate feeding regimes can be identified which correspond to different subpopulations [4].

A promising approach is to combine these CFD lifelines with intracellular mechanistic models, considering both the bioreactor and cell factory as the modeled system [152]. Such multiscale modeling can bring online bioprocess monitoring to the next level to, for instance, suggest how oxygen and biomass concentration influence ethanol synthesis in different fermenter locations [153,154,155]. Nonetheless, the current implementations consist of highly simplified, phenomenological *Penicillium chrysogenum* and yeast models [156,157]. A way to improve the quality of the simulations is to use state of the art kinetic CCM models [16,17,18,20], provided that they include variables that link cell physiology to the bioreactor environment. Finally, combining these CFD and mechanistic models with intracellular cell-to-cell heterogeneity and the growing importance of industrially relevant digital bioprocess twins [158,159] could enrich the predictability of these multiscale models to an unprecedented level.

## 10. Conclusions

Multiple models have been developed to understand how *Saccharomyces cerevisiae* navigates through glucose perturbations. Despite this, our understanding of the interplay between these dynamic environmental conditions and central carbon metabolism is still limited. This review aims at determining which have been the advances, limitations, and opportunities in *S. cerevisiae* CCM kinetic modeling. Our kinetic models have improved. Notably, it was first understood that glycolysis regulated the process largely in isolation, but more regulation layers have been considered to be new data made them observable.

Future research should focus on areas which current models lack but where understanding can be improved. For instance, models need to become more complex to consider pathways in the CCM, cofactor kinetics, PTMs and compartmentation. Furthermore, new datasets recently generated can be used in model development and validation. To further validate models, information on physiological, process variables and different experimental setups can be considered. In addition, coupling in vivo parameter quantification with advances in experimental measurements will result in highly predictive models. Finally, single-cell measurement technologies will extend our models from representing an average cell to the population heterogeneity, considerably improving our capacity for predictive modeling of industrial bioprocesses.

The resulting CCM models will be of great use to both academia and industry once they consider the cellular context this pathway interacts with. For instance, process variables with fundamental physiological information such as biomass growth rate, oxygen uptake limitation, or process yields will prove crucial in this mission. Finally, a complete representation of internal CCM dynamics will be accomplished once the different pathways composing it and cofactor dynamics are simultaneously represented, and not restricted to glycolysis alone.

## 11. Methods

The collection of literature and consequent screening of relevant works in this study took place in a systematic fashion. The guidelines set out in the Cochrane Handbook for Systematic Review and Meta-Analyses [160] were adapted to our research focus, similar to their implementation in other disciplines [10,11,12]. The selected literature was then surveyed to create an inventory of experimental data from glucose perturbation experiments and *S. cerevisiae* CCM kinetic models. Simultaneously, this helped to identify the relevant trends in the field that are studied in this paper.

The steps to collect the bibliography used in this work are described in Table 4. A clear objective and search query were described early on. Searching for this query generated an extensive literature collection from which appropriate papers were screened for using inclusion and exclusion criteria. The resulting literature pool was read and ranked for relevance according to another set of criteria. From the most relevant works, a snowball and citation search were used to double check that no relevant works were missed.

In this process, appropriate tools for each step were used. The initial search query was implemented in the Scopus abstract and citation database. This initial bibliography was loaded into the Rayyan webapp [161] to efficiently screen for inclusion and exclusion criteria based on abstract reading in a systematic way. The selected literature collection was from there on analyzed in depth using Mendeley. Finally, the resulting bibliometric network was visualized with the VOSviewer software [162].

From the resulting selected articles, an inventory of existing experimental data and kinetic models was made. Experimental data sets of interest concerned experiments where external perturbations in nutrient concentrations caused intracellular metabolomic or fluxomic changes in CCM. The models of interest for this work were mechanistic models with time-dependent description of intracellular concentrations of CCM. For the purpose of this research, not all data and models were downloaded, but when needed, data were acquired through the supplementary materials or by direct contact with the corresponding author. Models were downloaded from the BioModels [9] or JWS online databases [163] (exceptions of works prior to 2000). Recent experimental data could be downloaded online, to obtain datasets prior to 2010, it was often the case that the authors had to be personally contacted.

## Figures and Tables

**Table 4:** Steps followed to collect the literature used in this review.

Step	Description
1. Development of a search query	A search query was designed and implemented in the Scopus database document search. The time range selected was 2000–2020 to obtain a workable library size and relevant to the publication time. This query aimed to find all papers relevant to kinetic metabolic models of *S. cerevisiae*. Areas of uncertainty in models was an area of focus as well. The search query is: (TITLE-ABS-KEY (kinet* OR dynam* OR biochem*) AND TITLE-ABS-KEY (metabol*) AND TITLE-ABS-KEY (model* OR network*) AND TITLE-ABS-KEY (yeast OR “baker’s yeast” OR cerevisiae) AND TITLE-ABS-KEY ((paramet* OR structur* OR topolog* OR “in vivo” OR “in vitro”) AND (uncertain* OR sensitiv* OR crosstalk OR burden OR likelih* OR control OR energ* OR ptm OR transcription* OR translation* OR regulat* OR interact* OR multilevel)) OR TITLE-ABS-KEY ((paramet* OR structur* OR topolog* OR “in vivo” OR “in vitro” OR regulat* OR interact* OR multilevel) AND (uncertain* OR sensitiv* OR crosstalk OR burden OR likelih* OR control OR energ* OR ptm OR transcription* OR translation*))) AND DOCTYPE (ar OR re) AND PUBYEAR > 1999.
2a. Literature screening strategy: title and abstracts	The first screening round was performed using the RAYYAN webapp. Inclusion and exclusion criteria were used to determine if an article would be considered or not for our research. Since the library at this point was extensive (>3000 papers) and many articles had little relationship with our field, this step was performed only based on reading abstracts. Inclusion, exclusion, and undecided criteria were the following: Inclusion criteria: (1) Geographic location: no limitation, (2) Language: English, (3) Experimental scale: no limitation, (4) Publication type: article or reviews, (5) Organism: *Saccharomyces cerevisiae*, aka yeast, (6) Kinetic modeling, (7) Theoretical or experimental modeling, (8) Organelles: cytosol and mitochondria, (9) Yeast dynamic models external, but tightly related, to CCM and (10) State of the art yeast GSM of CCM. Exclusion criteria: (1) Non-peer review articles, (2) No patents, (3) Before 2000, (4) Mixed culture, (5) Not submerged growth, (6) Metabolic routes outside CCM, (7) Unconfined environment, (8) No modeling work and (9) Article duplicates.
2b. Literature screening strategy: content	The second round of screening took place in the Mendeley environment. The manuscripts that priorly fitted in the ‘inclusion’ group were read (in this case, not constrained to abstract only) to find if their main work focus was a dynamic metabolic model of CCM. From these collection, unique models were identified.
3. Extraction of relevant information	The following relevant information was extracted from each model: (1) Motivation/Research question, (2) Outcome of the research, (3) Future research proposed, (4) Type of dynamic modeling used, (5) Coverage of the model, (6) Presence of reaction that connect CCM to the remained of the metabolic network, (7) Modeling of dynamic and/or steady-state conditions, (8) Parameter values origin and (9) Presence or not of experimental data.
4. Quality assessment	To rank the relevance of the found models to our research, the following quality aspects were evaluated: (1) New knowledge to the understanding of *S. cerevisiae* glycolysis provided, (2) Extensive coverage of glycolysis and other pathways in CCM, (3) Inclusion of relevant variables external to CCM stoichiometry and kinetics (i.e., cofactor kinetics, sink reactions or post-translational regulation), (4) Detail in kinetic descriptions: from simple mass actions to more complex Michaelis–Menten kinetics with allosteric regulation, (5) Source of parameters in the model: experimental parameter measurements determined in conditions that do not resemble the cytosol (in vitro-like) are the least relevant. When conditions resemble the cytosol (in vivo-like) or parameters were estimated to fit the experimental metabolomics data, these are deemed as more relevant, (6) Validation with experimental data: the more variables and experimental setups used for validation, the better, and (7) Since models often build on top of each other, these often results in the most relevant models being the most complete.
5. Extra literature search	To check that no relevant literature was missed, *S. cerevisiae* CCM kinetic models were also searched for in the BioModels and the JWS databases. Furthermore, citation and snowball literature search were applied on the publications which contained the relevant and unique models.

## Abbreviations

The following abbreviations are used in this manuscript:

CCM	Central Carbon Metabolism
ODE	Ordinary Differential Equation
FAIR	Findability, Accessibility, Interoperability, and Reuse
TCA	TriCarboxylic Acid
GP	Glucose Perturbation
PPP	Pentose Phosphate Pathway
SS	Steady State
FF	Feast–Famine
HXK	Hexokinase
PYK	Pyruvate Kinase
FBP	Fructose-1,6-bis-phosphate
PFK	Phosphofructokinase
PKA	Protein Kinase A
PTM	Post-Translational Modification
GLT	Glucose Transporter
O_2_	Oxygen
CO_2_	Carbon Dioxide
GAPDH	Glyceraldehyde 3-phosphate dehydrogenase
NMR	Nuclear Magnetic Resonance
MS	Mass Spectroscopy
MLE	Maximum Likelihood Estimation
CFD	Computational Fluid Dynamics
